# Hypokalemia After Rituximab Administration in Steroid-Dependent Nephrotic Syndrome: A Case Report

**DOI:** 10.3389/fphar.2020.00915

**Published:** 2020-06-17

**Authors:** Francesco Guzzi, Mattia Giovannini, Carmela Errichiello, Giulia Liccioli, Francesca Mori, Rosa Maria Roperto, Paola Romagnani

**Affiliations:** ^1^Nephrology and Dialysis Unit, Department of Pediatrics, Meyer Children's University Hospital, Florence, Italy; ^2^Department of Biomedical Experimental and Clinical Sciences “Mario Serio”, University of Florence, Florence, Italy; ^3^Allergy Unit, Department of Pediatrics, Meyer Children's University Hospital, Florence, Italy

**Keywords:** adverse drug reactions, kalemia, biologic drugs, nephrology, case report

## Abstract

The monoclonal antibody rituximab is a commonly used steroid sparing agent for steroid-dependent idiopathic nephrotic syndrome of childhood. With this brief report, we describe the first case of symptomatic hypokalemia after intravenous rituximab administration in a young woman. The sudden onset of dizziness and palpitation prompted acute life-threatening hypokalemia recognition by blood gas analysis and electrocardiography. Her symptoms were rapidly controlled by intravenous potassium administration. Such adverse drug reactions, when mild and self-limiting, can easily be overlooked if not expected or investigated. Health professionals should take into account the possibility of acute hypokalemia after rituximab administration in order to promptly setup the appropriate treatment and limit potentially severe complications.

## Introduction

Idiopathic nephrotic syndrome (NS) is a relatively common disease of childhood, and the most frequent glomerular disease in children (Noone et al., 2018). NS is characterized by nephrotic range proteinuria (> 50 mg/kg/die), hypoalbuminemia (serum albumin < 2.5 g/dL), peripheral edema, and hyperlipidemia. The vast majority of NS patients are steroid-sensitive, with complete disease remission after steroid treatment, although many of them relapse. Approximately half of steroid-sensitive patients relapse many times per year, and nephrologists categorize them in “frequently relapsing” and “steroid-dependent”. These patients are at major risk for chronic steroid-related side effects (i.e. metabolic, ocular and skeletal complications) and substantial morbidity (Fakhouri et al., 2003; Rheault, 2016). For this reason, a wide range of steroid sparing agents such as levamisole, cyclophosphamide, cyclosporine A, tacrolimus, mycophenolate mofetil, and rituximab have been proposed over time (Noone et al., 2018), the latter being successfully used as steroid sparing agent in idiopathic NS in the last decade (Ravani et al., 2011; Iijima et al., 2014).

Rituximab is a chimeric mouse/human monoclonal antibody produced through genetic engineering techniques with the structure of a IgG1 kappa immunoglobulin and an molecular weight of about 145 kD. The transmembrane antigen CD20, located on pre-B and mature B lymphocytes is the specific target of this monoclonal antibody, which is capable to lead to B cell lysis through apoptosis and different immune mechanisms, including antibody-dependent cellular cytotoxicity and complement-dependent cytotoxicity. For this reason, rituximab is considered a key option to intervene upon B cells critical roles in inflammatory, such as cytokine production, antibodies production, antigen presentation, T-cells activation, proliferation and neoplastic processes, such as growth, anaplasia, and invasion. Specifically, rituximab is licensed for the treatment of a plenty of clinical conditions in adults and children, mainly neoplastic diseases, such as non-Hodgkin's lymphoma, chronic lymphocytic leukemia and inflammatory diseases, such as rheumatoid arthritis, granulomatosis with polyangiitis, microscopic polyangiitis, pemphigus vulgaris. Rituximab is administered as an intravenous infusion by an experienced healthcare professional with a specific background and in a clinical environment appropriate to manage adverse reactions to the drug, with a potentially prompt access to resuscitation facilities. Premedication with antipyretic and diphenhydramine therapies should be always administered before the infusion [Mabthera EPAR – Product Information. European Medicines Agency (internet). Available from: https://www.ema.europa.eu/en/documents/product-information/mabthera-epar-product-information_en.pdf; Rituxan labelling. Food and Drug Administration. Available from: https://www.accessdata.fda.gov/drugsatfda_docs/label/2020/103705s5461lbl.pdf – cited 2020 Mar 01]. A rich body of literature addressing acute and chronic adverse events following rituximab administration is available, also in the particular setting of idiopathic nephrotic syndrome (Kallash et al., 2019). However, to our knowledge, this is the first report of acute hypokalemia with clinical manifestations after rituximab administration.

## Case Report

**Figure 1:**
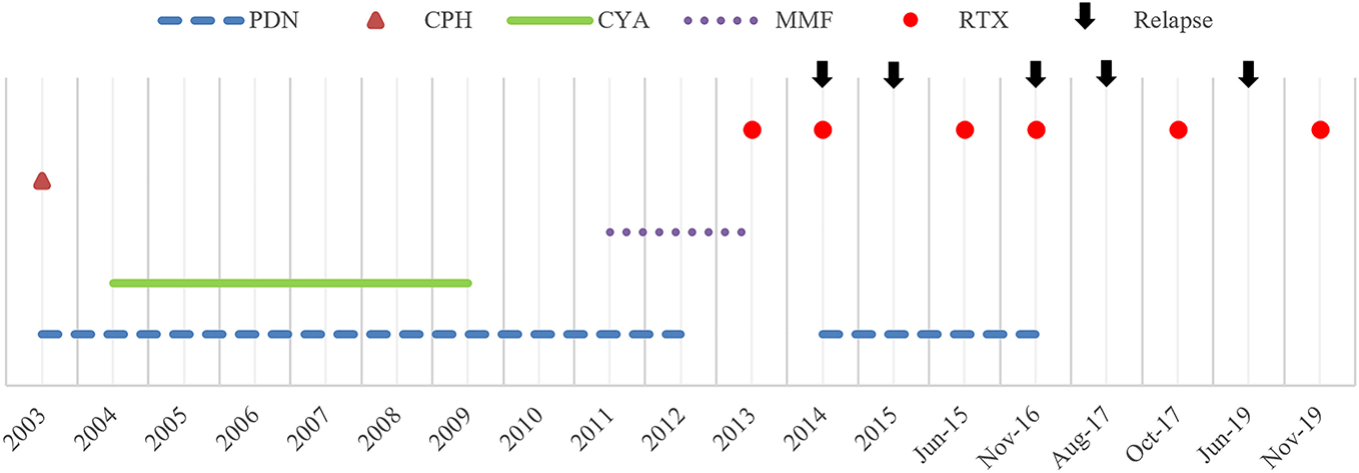
Historical view of immunosuppressive treatment with a detail on rituximab administrations and subsequent relapses. PDN, prednisone; CPH, cyclophosphamide; CYA, cyclosporine A; MMF, mycophenolate mofetil; RTX, rituximab.

A 4-year-old child of Moroccan origin, with non-consanguineous parents, was first diagnosed with idiopathic nephrotic syndrome in 2002. Since then, she was followed by the Nephrology Unit of the Meyer Children's University Hospital in Florence. Despite sensitive to steroid therapy, she soon developed prednisone dependence, and steroid sparing immunosuppressive drugs were administered (**Figure 1**).

However, steroid-free therapeutic regimen was hardly achieved, and the girl developed more than one steroid-related side effect; short stature, osteoporosis (vertebral collapse, with the need for a lumbosacral corset), infections (pneumonia) and gastrointestinal side effects were reported. For these reasons, the impossibility to wean from steroids, and the growing evidence in the literature, on March 2013, aged 15, she was started on rituximab, with a first intravenous infusion of 375mg/m^2^. Since then, she was able to rapidly taper and stop steroid therapy for almost one year. As expected (Sellier-Leclerc et al., 2012), she experienced subsequent relapses, with the need for new rituximab administrations, approximately one per year (**Figure 1**). From a retrospective review of clinical charts, no serious adverse events were ever reported following the monoclonal antibody administration. However, in two occasions, after rituximab administration in November 2016 and August 2017 (aged 18) the girl reported dizziness, hypotension and pre-syncopal symptoms, which were considered not clinically relevant.

**Figure 2:**
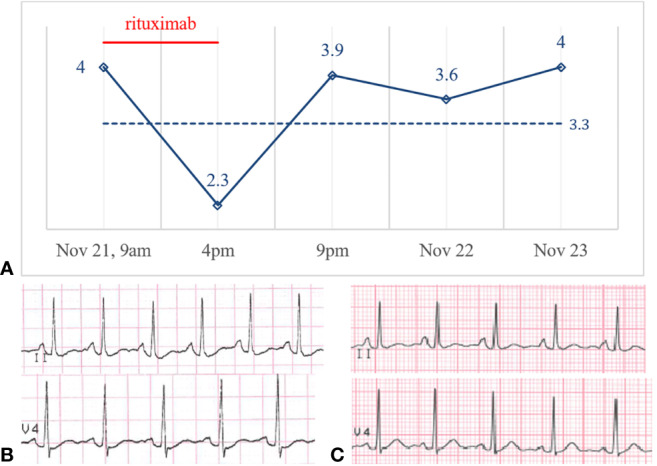
**(A)** Potassium levels (mmol/L) over time and potassium central laboratory normal value (3.3 mmol/L, dotted line). ECG before **(B)** and after **(C)** rituximab administration on November 21 and November 22, 2019.

On November 21, 2019, the girl was scheduled for her 6^th^ intravenous rituximab infusion (375mg/m^2^, total dose 500mg), in our Nephrology Unit, as an outpatient. Before starting the infusion, at 9am, a blood gas test was performed: her serum potassium level was 4.0 mmol/L (**Figure 2**)—sodium, calcium, magnesium, glucose, pH and bicarbonate levels were normal. After 3 hours from the beginning of the infusion the girl reported dizziness and palpitation, her heart rate increased from 60 to 100 bpm and the infusion was temporary stopped. At 4pm, about 7 hours from the beginning, the infusion was ended, but the girl was still symptomatic for tachycardia. A second blood gas test was then obtained, and potassium level was 2.3 mmol/L, with no significant changes in electrolytes, acid-base balance or other parameters. Hypokalemia was rapidly confirmed by a third blood gas analysis (2.4 mmol/L) and by the hospital central laboratory (2.5 mmol/L). An ECG was obtained reporting hypokalemia signs: normal sinus rhythm, HR 120 bpm, diffuse repolarization abnormalities, and not measurable QTc interval (**Figure 2**). Intravenous potassium (total dose K^+^ = 20 mmol) was started and the patient admitted to the pediatric ward for overnight monitoring. At 9pm, less than 5 hours from the end of the infusion, her symptoms were completely resolved, and her potassium level was back in the normal range (3.9 mmol/L, hospital central laboratory). The next morning, without any other supplementation, serum potassium level was 3.6 mmol/L, and the day after 4.0 mmol/L. A second ECG obtained on November 22 showed complete reversion of ventricular abnormalities (**Figure 2**). At follow-up the patient serum potassium levels were stably in the normal range.

## Discussion

Rituximab is a successful steroid sparing option in the case of idiopathic NS, given its action on B cells, that have recently been re-evaluated in the pathogenesis of this clinical condition (Colucci et al., 2016; Kallash et al., 2019). A wide range of data about adverse events associated with rituximab have been published, coming from clinical trials and post-marketing surveillance comprising patients with inflammatory or neoplastic diseases. Infusion-related adverse reactions are the most frequently observed events with clinical manifestations predominantly comprising fever, chills, asthenia, headache, anaphylaxis, anaphylactoid events, bronchospasm, respiratory distress, skin rash, urticaria, angioedema, or pruritus. These clinical manifestations are typically related to the first infusion and usually with an onset time of about 30-120 minutes. Given the rising use of monoclonal antibodies to treat several clinical conditions, a parallel increase in hypersensitivity reactions to these drugs have been recorded in the literature, including rituximab (Galvão and Castells, 2015; Wong and Long, 2017). They have been defined as belonging to different phenotypes associated to several endotypes: type I-like (IgE and non-IgE), cytokine release, mixed (type I-like and cytokine release) or type IV reactions (Isabwe et al., 2018). In the case of a confirmed IgE mediated reaction, desensitization with the drug, including rituximab, has demonstrated to be effective, safe and it is considered the first-line therapy in selected patients (Castells et al., 2008; Caimmi et al., 2014; Sloane et al., 2016; Diaferio et al., 2020).

Early- and late-onset hypogammaglobulinemia, neutropenia, and infections, including bacterial, fungal and viral (new and reactivated) episodes, are also commonly reported in association with rituximab administration (Kronbichler et al., 2017; Colucci et al., 2019). Hyperglycemia and cardiovascular disorders (mostly arrhythmias, hypertension, and hypotension) are associated with the drug administration as well. In very rare cases, rituximab has been associated to progressive multifocal leukoencephalopathy [Mabthera EPAR – Product Information. European Medicines Agency (internet). Available from: https://www.ema.europa.eu/en/documents/product-information/mabthera-epar-product-information_en.pdf; Rituxan labelling. Food and Drug Administration. Available from: https://www.accessdata.fda.gov/drugsatfda_docs/label/2020/103705s5461lbl.pdf – cited 2020 Mar 01].

Hypokalemia caused by rituximab administration has never been reported in the literature so far. Hypokalemia was reported as a rare adverse event of complex chemo-immunotherapy combinations for lymphoma treatment (Polprasert et al., 2011; Budde et al., 2013; Johnston et al., 2016; Godfrey et al., 2019; Shimada et al., 2020). However, none of these reports suggested a direct link between rituximab administration and hypokalemia. In particular, the addition of lenalidomide, everolimus, or vorinostat to classic R-CHOP (rituximab-cyclophosphamide, doxorubicin, vincristine, prednisone) or R-CVP (rituximab-cyclophosphamide, vincristine, prednisone) regimens, or in alternative therapy-induced diarrhea, were suggested to explain hypokalemia. In a recent analysis of the American National Cancer Institute, hypokalemia was reported as the most frequent metabolic disturbance after administration of many anticancer targeted therapies, but not rituximab (Jhaveri et al., 2016). As an example, treatment with monoclonal antibodies targeting the epidermal growth factor receptor-1 (EGFR-1 or HER-1), like cetuximab and panitumumab, was linked to hypokalemia (Jhaveri et al., 2017). In the kidney, EGFR regulates the transport of transient receptor potential melastatin (TRPM) 6/7 ion channels to the apical membrane of the distal renal tubule, thus regulating magnesium reabsorption. By blocking the EGFR, these drugs reduce apical TRPM ion channels, causing renal magnesium wasting and hypomagnesemia which may lead to hypokalemia and cardiac arrhythmias (Jhaveri et al., 2017). In one report of pemphigus patients treated with rituximab, an increase in KCNN4 potassium channels on B cell surface has been described (Caillot et al., 2018). However, B cell KCNN4 channels promote calcium influx in exchange with potassium efflux, not likely being responsible for hypokalemia.

Although limited by being a single case report, and by the impossibility to establish a precise mechanism and causal relationship, the acute, symptomatic, and promptly reversible hypokalemia experienced by our patient suggests, for the first time, a tight link with rituximab infusion. However, an immune-mediated hypersensitivity mechanism underlying the adverse reaction seems not likely. Moreover, the patient was completely fine in the previous days, her nephrotic syndrome was in remission phase, and she wasn't taking any other drug. Her magnesium and glucose levels were normal, both excluding a possible link with hypomagnesemia or abrupt hyperinsulinemia. More research data are needed about the action of rituximab on potassium ion channels in order to define the complete landscape of possible interactions. Frequently reported nonspecific symptoms, like palpitations and dizziness, and cardiac complications, like arrhythmias, may potentially be related to undetected hypokalemia. Although never documented in the patient history, we could speculate that acute hypokalemia could explain the symptoms she experienced also after prior infusions, highlighting how such adverse drug reaction, when mild and self-limiting, can easily be overlooked if not expected or investigated.

In conclusion, health professionals should take into account the possibility of acute hypokalemia after rituximab administration in the case of suggestive clinical manifestations, in order to promptly setup the appropriate treatment and avoid potentially serious complications.

## Data Availability Statement

All datasets presented in this study are included in the article/supplementary material.

## Ethics Statement

Written informed consent was obtained from the individual(s) for the publication of any potentially identifiable images or data included in this article.

## Author Contributions

FG and MG conceptualized, designed the research, acquired, analyzed, interpreted the data, drafted, and critically revised the manuscript. CE, GL, FM, RR, and PR analyzed, interpreted the data, and critically revised the manuscript. All authors contributed to the article and approved the submitted version.

## Conflict of Interest

The authors declare that the research was conducted in the absence of any commercial or financial relationships that could be construed as a potential conflict of interest.

## References

[B1] Budde L. E. Zhang M. M. Shustov A. R. Pagel J. M. Gooley T. A. Oliveira G. R. (2013). A phase I study of pulse high-dose vorinostat (V) plus rituximab (R), ifosphamide, carboplatin, and etoposide (ICE) in patients with relapsed lymphoma. Br. J. Haematol. 161, 183–191. 10.1111/bjh.12230 23356514 PMC3618618

[B2] Caillot F. Derambure C. Berkani N. Riou G. Maho-Vaillant M. Calbo S. (2018). Long-Term Increase of Kcnn4 Potassium Channel Surface Expression on B Cells in Pemphigus Patients after Rituximab Treatment. J. Invest. Dermatol. 138, 2666–2668. 10.1016/j.jid.2018.05.034 30472996

[B3] Caimmi S. M. E. Caimmi D. Riscassi S. Marseglia G. L. (2014). A New Pediatric Protocol for Rapid Desensitization to Monoclonal Antibodies. Int. Arch. Allergy Immunol. 165, 214–218. 10.1159/000369299 25531534

[B4] Castells M. C. Tennant N. M. Sloane D. E. Ida Hsu F. Barrett N. A. Hong D. I. (2008). Hypersensitivity reactions to chemotherapy: Outcomes and safety of rapid desensitization in 413 cases. J. Allergy Clin. Immunol. 122, 574–580. 10.1016/j.jaci.2008.02.044 18502492

[B5] Colucci M. Carsetti R. Cascioli S. Casiraghi F. Perna A. Ravà L. (2016). B Cell Reconstitution after Rituximab Treatment in Idiopathic Nephrotic Syndrome. J. Am. Soc Nephrol. 27, 1811–1822. 10.1681/ASN.2015050523 26567244 PMC4884116

[B6] Colucci M. Carsetti R. Serafinelli J. Rocca S. Massella L. Gargiulo A. (2019). Prolonged Impairment of Immunological Memory After Anti-CD20 Treatment in Pediatric Idiopathic Nephrotic Syndrome. Front. Immunol. 10, 1653. 10.3389/fimmu.2019.01653 31379849 PMC6646679

[B7] Diaferio L. Giovannini M. Clark E. Castagnoli R. Caimmi D. (2020). Protocols for drug allergy desensitization in children. Expert Rev. Clin. Immunol. 16, 91–100. 10.1080/1744666X.2019.1698294 31771366

[B8] Fakhouri F. Bocquet N. Taupin P. Presne C. Gagnadoux M.-F. Landais P. (2003). Steroid-sensitive nephrotic syndrome: from childhood to adulthood. Am. J. Kidney Dis. 41, 550–557. 10.1053/ajkd.2003.50116 12612977

[B9] Galvão V. R. Castells M. C. (2015). Hypersensitivity to biological agents-updated diagnosis, management, and treatment. J. Allergy Clin. Immunol. Pract. 3, 175–185; quiz 186. 10.1016/j.jaip.2014.12.006 25754718

[B10] Godfrey J. K. Nabhan C. Karrison T. Kline J. P. Cohen K. S. Bishop M. R. (2019). Phase 1 study of lenalidomide plus dose-adjusted EPOCH-R in patients with aggressive. Cancer 125, 1830–1836. 10.1002/cncr.31877 30707764

[B11] Iijima K. Sako M. Nozu K. Mori R. Tuchida N. Kamei K. (2014). Rituximab for childhood-onset, complicated, frequently relapsing nephrotic syndrome or steroid-dependent nephrotic syndrome: a multicentre, double-blind, randomised, placebo-controlled trial. Lancet 384, 1273–1281. 10.1016/S0140-6736(14)60541-9 24965823

[B12] Isabwe G. A. C. Garcia Neuer M. de Las Vecillas Sanchez L. Lynch D.-M. Marquis K. Castells M. (2018). Hypersensitivity reactions to therapeutic monoclonal antibodies: Phenotypes and endotypes. J. Allergy Clin. Immunol. 142, 159–170.e2. 10.1016/j.jaci.2018.02.018 29518427

[B13] Jhaveri K. D. Sakhiya V. Wanchoo R. Ross D. Fishbane S. (2016). Renal effects of novel anticancer targeted therapies: a review of the Food and Drug Administration Adverse Event Reporting System. Kidney Int. 90, 706–707. 10.1016/j.kint.2016.06.027 27521117

[B14] Jhaveri K. D. Wanchoo R. Sakhiya V. Ross D. W. Fishbane S. (2017). Adverse Renal Effects of Novel Molecular Oncologic Targeted Therapies: A Narrative Review. Kidney Int. Rep. 2, 108–123. 10.1016/j.ekir.2016.09.055 29318210 PMC5720524

[B15] Johnston P. B. LaPlant B. McPhail E. Habermann T. M. Inwards D. J. Micallef I. N. (2016). Everolimus combined with R-CHOP-21 for new, untreated, diffuse large B-cell lymphoma (NCCTG 1085 [Alliance]): safety and efficacy results of a phase 1 and feasibility trial. Lancet Haematol. 3, e309–e316. 10.1016/S2352-3026(16)30040-0 27374464 PMC4958393

[B16] Kallash M. Smoyer W. E. Mahan J. D. (2019). Rituximab Use in the Management of Childhood Nephrotic Syndrome. Front. Pediatr. 7, 178. 10.3389/fped.2019.00178 31134169 PMC6524616

[B17] Kronbichler A. Windpessl M. Pieringer H. Jayne D. R. W. (2017). Rituximab for immunologic renal disease: What the nephrologist needs to know. Autoimmun Rev. 16, 633–643. 10.1016/j.autrev.2017.04.007 28414152

[B18] Noone D. G. Iijima K. Parekh R. (2018). Idiopathic nephrotic syndrome in children. Lancet 392, 61–74. 10.1016/S0140-6736(18)30536-1 29910038

[B19] Polprasert C. Wongjitrat C. Wisedopas N. (2011). Case report: severe CMV colitis in a patient with follicular lymphoma after chemotherapy. J. Med. Assoc. Thai 94, 498–500. 21591537

[B20] Ravani P. Magnasco A. Edefonti A. Murer L. Rossi R. Ghio L. (2011). Short-Term Effects of Rituximab in Children with Steroid- and Calcineurin-Dependent Nephrotic Syndrome: A Randomized Controlled Trial. Clin. J. Am. Soc Nephrol. 6, 1308–1315. 10.2215/CJN.09421010 21566104 PMC3109926

[B21] Rheault M. N. (2016). Nephrotic Syndrome: Updates and Approaches to Treatment. Curr. Treat. Options Pediatr. 2, 94–103. 10.1007/s40746-016-0044-x

[B22] Sellier-Leclerc A.-L. Baudouin V. Kwon T. Macher M.-A. Guerin V. Lapillonne H. (2012). Rituximab in steroid-dependent idiopathic nephrotic syndrome in childhood–follow-up after CD19 recovery. Nephrol. Dial. Transplant. 27, 1083–1089. 10.1093/ndt/gfr405 21810762

[B23] Shimada K. Yamaguchi M. Atsuta Y. Matsue K. Sato K. Kusumoto S. (2020). Rituximab, cyclophosphamide, doxorubicin, vincristine, and prednisolone combined with high-dose methotrexate plus intrathecal chemotherapy for newly diagnosed intravascular large B-cell lymphoma (PRIMEUR-IVL): a multicentre, single-arm, phase 2 trial. Lancet Oncol. 21, 593–602. 10.1016/S1470-2045(20)30059-0 32171071

[B24] Sloane D. Govindarajulu U. Harrow-Mortelliti J. Barry W. Hsu F. I. Hong D. (2016). Safety, Costs, and Efficacy of Rapid Drug Desensitizations to Chemotherapy and Monoclonal Antibodies. J. Allergy Clin. Immunol. Pract. 4, 497–504. 10.1016/j.jaip.2015.12.019 26895621

[B25] Wong J. T. Long A. (2017). Rituximab Hypersensitivity: Evaluation, Desensitization, and Potential Mechanisms. J. Allergy Clin. Immunol. Pract. 5, 1564–1571. 10.1016/j.jaip.2017.08.004 29122155

